# Associating herpes zoster ophthalmicus with cerebral vasculitis

**DOI:** 10.1590/0037-8682-0155-2023

**Published:** 2023-07-24

**Authors:** Jean Levi Ribeiro de Paiva, Tânia Aparecida Marchiori de Oliveira Cardoso, Fabiano Reis

**Affiliations:** 1 Universidade Estadual de Campinas, Departamento de Anestesiologia, Oncologia e Radiologia, Campinas, SP, Brasil. Universidade Estadual de Campinas Departamento de Anestesiologia, Oncologia e Radiologia Campinas SP Brasil; 2 Universidade Estadual de Campinas, Departamento de Neurologia, Campinas, SP, Brasil. Universidade Estadual de Campinas Departamento de Neurologia Campinas SP Brasil

A 15-year-old boy presented with a fever that lasted 50 days, with right eyelid edema and hyperemia, and a visual deficit diagnosed as chorioretinitis. One month later, the patient developed a brief episode of left hemiparesthesia and, subsequently, sudden paresis of the left upper and lower limbs without skin lesions. Cerebrospinal fluid (CSF) analysis revealed pleocytosis with a predominance of lymphocytes, restricting the differential diagnosis to viral infections, syphilis, inflammatory and autoimmune disease. The latter three were deemed unlikely after extensive laboratory analyses.

**FIGURE 1:**
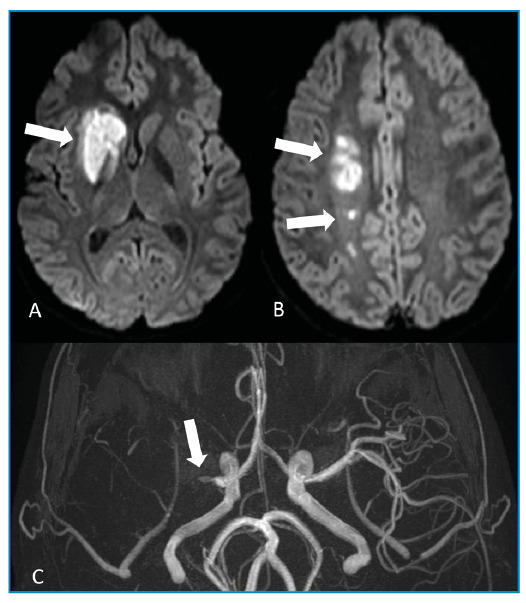
**A and B.** Axial diffusion-weighted images (DWI) showing right basal ganglia, internal capsule, and deep white matter hyperintensity (arrows), which had corresponding hypointensity on the apparent diffusion coefficient (ADC) map (not shown). This indicates restricted diffusion, consistent with acute ischemic stroke. **C.** Three-dimensional time-of-flight magnetic resonance angiography (3D-TOF) showing the absence of a flow signal in the right middle cerebral artery starting from its origin (arrow).

**FIGURE 2:**
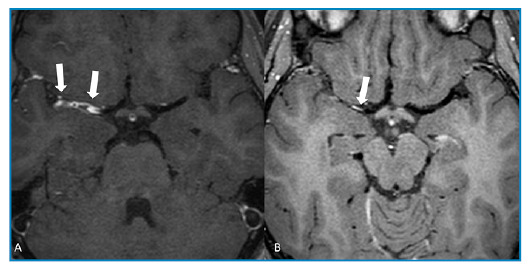
**A.** Post-contrast axial high-resolution vessel wall imaging (HR-VWI) showing concentric irregular wall thickening and enhancement of segments M1 and M2 of the right middle cerebral artery, consistent with vasculitis (arrows). **B.** Post-contrast axial HR-VWI 17 months after antiviral treatment, showing a marked reduction in vessel wall thickening, with slight wall enhancement, possibly related to sequelae of the infection (arrow).

Magnetic resonance imaging (MRI) revealed lesions in the right basal ganglia, internal capsule, and deep white matter with restricted diffusion, consistent with acute ischemic stroke (Figure 1)**.** Three-dimensional time-of-flight magnetic resonance angiography showed a lack of flow signal in the right middle cerebral artery (Figure 1), and high-resolution vessel wall imaging (HR-VWI) revealed concentric wall thickening with contrast enhancement in the right M1 and M2 segments, indicating vasculitis (Figure 2)**.** Subsequent CSF analysis revealed elevated levels of IgG for the varicella zoster virus, and the patient was treated with acyclovir for 14 days. After treatment, HR-VWI showed a marked reduction in wall enhancement (Figure 2)**.**

Herpes zoster ophthalmicus is associated with vasculopathy and cerebral infarction and has significant morbidity and mortality when untreated. Consequently, swift diagnosis is crucial but can be very challenging, especially in the absence of typical cutaneous lesions. In this context, MRI is a useful tool for diagnosing stroke and observing infectious vasculitis^1^^,^^2^^,^^3^. 
